# Rasgrp1-Mediated Dampening of EGFR Signals Supports Coordinated Mammary Gland Development

**DOI:** 10.3390/cells15141288

**Published:** 2026-07-18

**Authors:** Alexandr Samocha, Lauren Shechtman, Eunice Bonfil Tapia, Hanna M. Doh, Oghenekevwe Gbenedio, Vaishnavi Sitarama, Quy H. Nguyen, Joshua D. Rudolph, Walter L. Eckalbar, Andrea J. Barczak, Yi Miao, Philippe Depeille, K. Christopher Garcia, Devon A. Lawson, Kai Kessenbrock, Jeroen P. Roose

**Affiliations:** 1 Department of Anatomy, University of California San Francisco, 513 Parnassus Avenue, Room HSW-1325, San Francisco, CA 94143, USA; ajsamocha@gmail.com (A.S.); lauren.shechtman@ucsf.edu (L.S.); 0330euniceb@berkeley.edu (E.B.T.); hanna.doh@gmail.com (H.M.D.); oghenekevwe.gbenedio@ucsf.edu (O.G.); vaishnavi.sitarama@gmail.com (V.S.); depeille.work@gmail.com (P.D.); 2 Department of Biological Chemistry, University of California Irvine, Irvine, CA 92697, USA; quyhn@uci.edu (Q.H.N.); kai.kessenbrock@uci.edu (K.K.); 3 UCSF Genomics CoLab, Lung Biology Center, School of Medicine, University of California San Francisco, San Francisco, CA 94143, USA; josh.d.rudolph@gmail.com; 4 UCSF Genomics CoLab, Department of Medicine, University of California San Francisco, San Francisco, CA 94143, USA; walter.eckalbar@ucsf.edu (W.L.E.); andrea.barczak@ucsf.edu (A.J.B.); 5 Department of Molecular and Cellular Physiology, Department of Structural Biology, Howard Hughes Medical Institute, Stanford University School of Medicine, Stanford, CA 94305, USA; yi.miao@stanford.edu (Y.M.); kcgarcia@stanford.edu (K.C.G.); 6 Department of Physiology and Biophysics, University of California Irvine, Irvine, CA 92697, USA; dalawson@uci.edu

**Keywords:** mammary gland, TEB, EGFR, stem and progenitor cells, cell fate, RasGEF, Rasgrp1, PI3 kinase, organoid, transplantation

## Abstract

**Highlights:**

**What are the main findings?**

- Rasgrp1 functions as a critical dampener of EGFR–Ras–PI3K–AKT signaling in mammary epithelial cells; its loss enhances proliferative signaling and enables aberrant EGF-driven branching and colony formation;
- EGFR signals effectively suppress Wnt-dependent stem cell programs in mammary organoids and *Rasgrp1* deficiency leads to impaired ductal elongation and persistent terminal end buds.

**What are the implications of the main findings?**

- Coordinated mammary gland development depends on precise calibration of EGFR signaling; Rasgrp1 acts as a critical rheostat of this signal;
- Disruption of this regulatory balance may represent a general mechanism underlying epithelial disorganization and tumorigenesis.

**Abstract:**

Mammary gland development during puberty requires tightly coordinated epithelial proliferation, lineage specification, and branching morphogenesis, processes critically regulated by growth factor signaling. While epidermal growth factor receptor (EGFR) signaling is essential for ductal development, how its activity is quantitatively controlled within mammary epithelial cells (MECs) remains incompletely understood. Here, we identify Rasgrp1, a Ras guanine nucleotide exchange factor, as a key modulator of EGFR signaling in the mammary epithelium. Using *Rasgrp1*-deficient mice, primary MEC assays, and organoid models, we demonstrate that loss of *Rasgrp1* leads to elevated EGFR–Ras–PI3K–AKT and mTORC1-S6 signaling, resulting in enhanced proliferative capacity and aberrant EGF-driven branching. Transcriptomic analysis of organoids reveals that EGF signaling suppresses Wnt/R-spondin-dependent stem-cell gene programs, suggesting that excessive EGFR activity disrupts stem cell maintenance. In vivo, *Rasgrp1* deficiency causes impaired ductal elongation, persistent terminal end buds, and increased epithelial proliferation, indicating a breakdown in the spatial and temporal coordination of mammary morphogenesis. Together, our findings establish Rasgrp1 as a signaling rheostat that dampens EGFR pathway activity to support coordinated mammary gland development. These results highlight the importance of precise signaling calibration in epithelial development and suggest broader implications for Ras pathway regulation in tissue homeostasis and disease.

## 1. Introduction

The mammary gland is a dynamic organ composed of a network of bilayered epithelial ducts that develop primarily during puberty. During the ductal phase in puberty, coordinated proliferation and branching morphogenesis transform a rudimentary embryonic structure into a mature ductal tree [1,2,3,4]. These ducts arise from terminal end buds (TEBs), which consist of an outer layer of cap cells surrounding an inner mass of body cells. Within this inner compartment, mammary stem cells and progenitors form a heterogeneous population [5] that gives rise to the bilayered ductal epithelium, a central luminal layer and a basal myoepithelial layer adjacent to the basement membrane [2,4].

The EGFR (epidermal growth factor receptor) family includes EGFR (ErbB1/Her1), ErbB2/Her2, ErbB3/Her3, and ErbB4/Her4, with ligands such as EGF, transforming growth factor-α (TGFα), and amphiregulin (AREG) [6,7]. Elegant tissue recombination and cleared fat pad transplantation studies demonstrated that stromal EGFR signaling is essential for ductal morphogenesis [8,9,10]. Proper EGFR signaling is also required in mammary epithelial cells (MECs) but remains incompletely defined. Genetic mouse models support a functional requirement for ErbB receptors in the epithelium: loss of *ErbB2* impairs ductal outgrowth [11,12], *ErbB3* deficiency reduces TEB size and increases branch density [13], and *ErbB4* deletion affects lactation but not pubertal development [14].

Growth factor receptors such as the EGFR activate Ras via Ras guanine nucleotide exchange factors (RasGEFs), triggering downstream pathways including PI3K (phosphatidylinositol-3 kinase) [15]. The RasGEFs SOS1 (Son of Sevenless 1) and RasGRP1 (Ras-guanine-releasing protein 1) are structurally distinct [16,17,18]. In lymphocytes, these two RasGEFs cooperate to enable high-amplitude Ras signals [19,20,21,22,23,24,25]. Rasgrp1 plays a promoting role in skin carcinogenesis [26,27]. In contrast, in colorectal carcinoma cells, RasGRP1 is a tumor suppressor and dampens proliferative EGFR–SOS1–Ras signals [28]. Rasgrp1 is expressed in normal epithelial cells in the intestine [28] and bladder [29], but detailed cell biological studies on the roles in these epithelial cell subsets are lacking.

We noted that pups from *Rasgrp1*-deficient moms remained relatively small until they switched to eating solid food. We mined publicly available cell-line datasets and noted expression of RasGRP1 in breast cancer cell lines [30]. These expression characteristics and the observations from our mouse colony suggested an unexpected role for Rasgrp1 in regulating mammary gland biology, prompting us to investigate its function in this tissue. We find that Rasgrp1 is expressed within mammary epithelial cells and acts as a previously unrecognized modulator of EGFR signaling in MECs. Using genetic models, primary MEC assays, and organoid systems, we investigated the function of Rasgrp1 in MEC EGFR signaling and downstream cellular responses. Loss of *Rasgrp1* leads to elevated EGFR–Ras–PI3K–AKT and mTORC1-S6 signaling, resulting in enhanced proliferative capacity and aberrant EGF-driven branching.

## 2. Materials and Methods

### 2.1. Mice

Mice were handled according to the Institutional Animal Care and Use Committee regulations, described in the Roose Laboratory of University of California, San Francisco (UCSF, San Francisco, CA, USA) mouse protocol AN84051, ‘Ras Signal Transduction in Lymphocytes and Cancer’. Mice were housed and treated in compliance with the Institutional Animal Care and Use 559 Committee (IACUC) guidelines at the University of California, San Francisco. Date of Approval 06 February 2026. Protocol: APPROVAL NUMBER: AN208395-00A; Tri-560 ennial Expiration Date: 25 August 2028. Rasgrp1 knockout (*Rasgrp1^−/−^*) mice have a T-cell development defect [31] and were provided by J. Stone, as described in Depeille et al. [28]. *Rasgrp1^−/−^* and wild-type (WT) mice were both on a C57BL/6 background. Mice were bred and used as controls. Constitutive Rasgrp1 knockout (*Rasgrp1^−/−^*) mice [31] displayed regular litter size, pup birth weight, sex ratio, and survival, but pups from *Rasgrp1^−/−^* dams appeared smaller during lactation.

### 2.2. Mammary Whole Mount and Carmine Stain

The protocol was adapted from Kouros-Mehr H. et al. [32]. Inguinal #4 mammary fat pads were removed carefully from experimental mice. Glands were spread on glass slides (Fisherbrand, Waltham, MA, USA, 12-550-15) and fixed overnight in 3:1 ethanol to glacial acetic acid. Slides were transferred to 70% EtOH then 50% ethanol for 10 min. Slides were washed with tap water slowly to remove ethanol. Slides were placed in new slide holders containing Carmine Red (carmine, Sigma, St. Louis, MO, USA, C1022; aluminum potassium sulfate, Sigma A7167). Mammary tissue was then transferred to 70% then 95% ethanol for 15 min and 100% ethanol for 15 × 3 min. Tissue was then transferred to Histo-Clear II (National Diagnostics, Atlanta, GA 30336, USA, HS-202) for 2 h and then placed into fresh Histo-Clear. Slides were imaged using a dissection scope. The number and location of terminal end buds were assayed. Image analysis software (ImageJ, 1.53c) was used to determine the length of mammary duct branching.

### 2.3. Single Mammary Epithelial Cell Prep for FACS

The single cell isolation procedure for FACS from mammary tissue was adopted from a protocol provided by Kessenbrock K. and Lawson D (University of California, Irvine, CA, USA). The #1–#5 fat pads were removed from 4- to 9-week-old mice and placed in a dry 10 cm dish. The tissue was chopped with a razor until slurry-like in consistency. Mammary tissue was shaken in collagenase medium (2 mg/mL collagenase type IV, Sigma C5138, in DMEM/F12, Corning, New York 14831, USA, 10-090) at 37 °C for 1 h. Digested tissue was spun down at 1500 rpm for 5 min and then washed with PBS. MECs are freed using 0.05% Trypsin/EDTA (Corning 25-0520), and excess DNA was removed using DNAse (Worthington, Lakewood, NJ 08701, USA, LS002139). For FACS experiments on mammary organoids, TrypLE Select (Life Technologies, Carlsbad, CA 92008, USA, 12563011) was added to wells containing organoids embedded in Matrigel pellets. Organoids were incubated at 37 °C for 10 min. After incubation, organoid suspension was pipetted vigorously 10–15 times to further dissociate organoids into single cells. The suspension was spun down at 1500 rpm for 5 min and then washed with PBS. Single MECs are counted and placed into FACS tubes (Corning, NY 14831, USA, Falcon, 352235).

### 2.4. Basal and Luminal FACS Stain on Primary Mouse MECs

We used flow cytometry to assess luminal (CD49f/EpCAM^hi^) and basal/mammary stem cell-containing CD49f^hi^/EpCAM populations. Single MECs were isolated from WT, Rasgrp1^−/−^, and Rasgrp1^Anaef^ mice and placed into 500 μL DMEM/F12 FACS tubes (Corning, NY 14831, USA, Falcon, 352235). Single color controls were made for CD49f, EpCAM, and Lin- (CD31, CD45, Ter119) in addition to a no-stain control. FACS tubes are placed at 4 °C in the dark for 20 min. Cells were spun down at 1500 rpm for 5 min and aliquoted into new FACS tubes through the cap filter. FACS tubes were kept on ice. FACS stains were performed on an LSRII and an FACS Aria II machine (BD Biosciences, San Joze, CA 95131, USA). Sytox blue (ThermoFisher Scientific, Waltham, MA 02451, USA, S3457) was added to Lin- and ALL tubes just prior to their run to differentiate lin-/live (MECs) from lin+/dead (stroma).

### 2.5. RNA Extraction and Real-Time PCR

Total RNA was isolated from total MECs and basal, luminal, and stromal cells using an RNeasy Kit (Qiagen, Hilden, Germany). RNA underwent reverse-transcription with random primers (Invitrogen, Carlsbad, CA, USA) and reverse transcriptase. RNA quantity and quality were assayed via NanoDrop spectrophotometer (ThermoFisher Scientific, Waltham, MA 02451, USA) 260/60/280. SensiMix II (Bioline, London, UK) was added to samples, made in triplicate, and placed in real-time (RT) PCR plates (Eppendorf, Hamburg, Germany, 951022015) with clear film (Eppendorf, 0030132947). RT PCR was performed using the RealPlex2 (Eppendorf). Expression was normalized to ß-actin (Mm02619580_g1, Applied Bio Systems, Foster City, CA, USA) and quantified using the CT comparison method. This method was detailed by Eppendorf. Rasgrp1 primer (Mm00448564_m1) was obtained from Applied Bio Systems.

### 2.6. Western Blot

Cells were placed in 6-well plates and starved for 2 h at 37 °C in PBS. After resting, cells were stimulated for 0, 2, 5, and 30 min in EGF. Cells were lysed with ice-cold NP40 with added protease inhibitors (10 mM sodium fluoride, 2 mM sodium orthovanadate, 0.5 mM EDTA, 2 mM phenylmethylsulphonyl fluoride, 1 mM sodium molybdate, aprotinin (10 mg/mL), leupeptin (10 mg/mL), and pepstatin (1 mg/mL)). After lysing for 30 min, lysate was centrifuged at 4 °C and resuspended in 2X sample buffer. Lysates were run on pre-cast NuPAGE^TM^ 4–12% Bis-Tris gel (NP0335BOX, Invitrogen, Carlsbad, CA, USA) and transferred to PVDF membranes. Protein was incubated with primary antibodies overnight. Western blots were visualized with enhanced chemo-luminescence substrate (Thermo Scientific, Waltham, MA 02451, USA, 32106) and imaged on a Fuji LAS 4000 image station (GE Healthcare, Chicago, IL, USA). Bands were quantified with Multi Gauge software (GE Healthcare, Chicago, IL, USA), and densitometry was determined within a linear range of the exposure. Values were normalized to loading controls. The intensity was reported as the fold difference from the control sample.

### 2.7. Antibodies

Primary antibodies were obtained from the following sources and used at the indicated concentrations: P-MEK 1/2 (1:250; 2338S), P-p44/42 (1:250; 9102S), p44/42 MAPK (1:250; 9102), P-AKT S473 (1:250; 4058L), AKT (1:250; 9272), P-S6 ribosomal protein S235/236 (1:300; 2211L), S6 ribosomal protein (1:300; 2317), and cleaved caspase-3 (1:300; 9661S) from Cell Signaling; Ki-67 (1:500; ab15580), Rasgrp1 (1:100; ab37927), and Cytokeratin 5 (1:100; ab52635) from Abcam; CD49f-PE-Cy7 (1:100; Invitrogen 25-0495-82); EpCAM-APC (1:63; Invitrogen 17-5791-82); CD31-450 (1:170; Invitrogen 48-0311-82); CD45-450 (1:170; Invitrogen 48-0451-82); and Ter119-450 (1:170; Invitrogen 48-5921-82) from eBioscience; α-tubulin (1:2000; T6074) from Sigma-Aldrich; Troma-1 cyokertain 8 (1:100; AB_531826) from Developmental Studies Hybridoma Bank; murine Rasgrp1 m199 from Depeille et al. (2015) [28]. Secondary antibodies were obtained from the following sources and used at the indicated concentrations: Alexa Fluor 448 (1:500 in Matrigel IF, 1:250 slide IF; A21206), Alexa Fluor 555 (1:500 in Matrigel IF, 1:250 slide IF; A21434), and Alexa Fuor 568 (1:500 in Matrigel IF, 1:250 slide IF; A21069).

### 2.8. 3D Mammary Epithelial Colony-Forming Cell Assay

MEC clusters were first obtained following the protocol described in this manuscript. MEC clusters were then suspended in 2 mL of 0.05% trypsin/EDTA and placed in the 37 °C incubator for 6 min. Cells were taken out every 2 min and pipetted up and down several times to assist in the separation of clusters into individual cells. A total of 5 mL of HBSS + 2% fetal bovine serum was added to stop trypsinization. Then, single cells were re-pelleted and filtered over a 100 µM strainer. MECs were counted for the colony-forming assay or frozen in DMEM/F12 + 50% FBS + 10% DMSO. For Eph4 cells, 0.25% trypsin/EDTA was used to detach cells from the plate and inspected for single cellularity. Approximately 20 µL Matrigel platforms were created in a 48-well plate and allowed to sit for 20 min at 37 °C to solidify. Isolated MECs were resuspended in growth-factor-reduced Matrigel to produce 2500 cells/20 µL. Twenty microliters of the Matrigel-cell suspension was added on top of the Matrigel platform in each well and placed in the 37 °C incubator for 20 min to solidify. Growth factor and inhibitor medium were then added. The growth factor media used was as follows: 2.5 nM FGF, 2.5 nM EGF, 1 µM gefitinib erlotinib, in 1x pen/strep, 1x insulin transferrin, and sodium selenite DMEM/F12. The size and number of the colonies of the organoids were monitored for up to 10 days.

### 2.9. Traditional Three-Dimensional Mammary Gland Matrigel Cultures

Four- to nine-week-old mice were selected for our experiments. CO_2_-euthanized mice were placed chest up and sprayed with 70% ethanol. We made a medial cut distally down the abdominal skin, which was peeled away from the peritoneum of the abdominal cavity. Inguinal (#4) fat pads were removed and cut with a scalpel until loosened. Mammary tissue and mammary epithelial cells (MECs) were transferred into 0.45 µM of filtered collagenase solution; DMEM/F12 (UCSF Cell Culture Facility, CCFAA010-167201), 5% FBS, 50 mg/mL gentamicin (Gibco, Grand Island, NY, USA, 83-50721M), 5 µg/mL insulin (Sigma, St. Louis, MO, USA, I5508), 2 mg/mL trypsin (Gibco, 27250-018), and type IV collagenase (Gibco,17104-019). Glands were shaken at 37 °C for 35 min. The tissue then underwent differential centrifugation and treatment with 2 U/µL DNAse (Sigma D4263-1VL) to isolate MEC clusters. Twenty microliters of growth-factor-reduced Matrigel (BD 354230) platforms were created in glass-bottom chamber slides (Lab-Tek^®^, Nunc, Roskilde, Denmark, 177379) or 24-well plates and incubated for 20 min at 37 °C to solidify. Organoid density was determined before suspending MEC clusters in growth-factor-reduced Matrigel at 2 organoids/µL. Twenty microliters of the Matrigel-cell suspension was added on top of the Matrigel platform in each well and placed in the 37 °C incubator for 20 min to solidify. Five hundred microliters of DMEM/F12 media was added after solidification. The growth factor media used was as follows: 2.5 nM FGF, 2.5 nM EGF, in 1x pen/strep, 1x ITS, 1x DMEM/F12. The growth and branching of organoids were monitored for up to 10 days. The inhibitor given at day 3 was as follows: EGFRi (10 µM Erlotinib). For organoids, Rsondin2 and NGS Wnt were added at the indicated concentrations.

### 2.10. Nuclear Counterstain of Organoids and Colony-Forming Assay

Nuclear counterstaining for visualization was conducted using Hoechst^®^ 33342 (Thermo Scientific, Waltham, MA 02451, USA, H1399) and followed the provided protocol. In short, Hoechst^®^ dye was diluted 1:2000 in PBS and added to MECs for 10 min, which were protected from the light. The staining solution was removed, and the cells were washed 3x with PBS. Images were captured using an inverted fluorescence phase contrast microscope (Keyence BZ-X710, BZX Viewing Software, Osaka, Japan).

### 2.11. RNAseq Data Analysis

STAR [33] version 2.7.0f was used to align reads to the Mouse genome, version GRCm38.96, no adapter clipping or filtering was performed before alignment. Reads uniquely mapped to the mouse genome were used to assess expression changes among genes using DESeq2 [34]. Variance-stabilized counts were used for creating the heatmap, while blind-stabilized counts were used for PCA. PCA was generated using the prcomp function from the stats (https://www.rdocumentation.org/packages/stats/versions/3.6.2 (accessed on 14 July 2026)) package in R. The Plotly (https://cran.r-project.org/web/packages/plotly/index.html (accessed on 14 July 2026)) R package was used for making 3D PCA plots. The mean stabilized read count of each gene was subtracted from all samples’ stabilized read counts for that gene to generate the color grading, and any differences larger than the 97.5th percentile were set to the 97.5th (0.5 variance-stabilized gene counts above the mean) percentile. Any stabilized gene count differences lower than the 2.5th percentile were similarly set to the 2.5th (−0.5 variance-stabilized gene counts below the mean) percentile.

### 2.12. Immunofluorescence of Mammary Whole Mount Sections

Mammary fat pads were dissected, placed on glass slides, fixed in 4% PFA for 1 h, and placed overnight in 30% sucrose overnight. Mammary tissue was placed in 1:1 OCT (Tissue-Tek, Torrance, CA, USA)/30% sucrose for 1 h. Tissue was embedded in OCT. Sections (15 µm thick, Leica CM 1950 Cryostat, Leica Biosystems, Wetzlar, Germany) were obtained, setting the cooling block to the maximum cold. The sections were dried at room temperature for 1 h, and then incubated in 0.3% Triton X100 (Sigma, St. Louis, MO, USA) in PBS. Slides were rinsed with PBS and blocked for 1 h (10% normal donkey serum, 3% bovine serum albumin, and 0.1% Triton X100 in PBS). Primary antibodies were diluted in blocking buffer, added to the tissue, and incubated overnight at 4 °C overnight. The slides were washed with PBS and incubated in secondary antibodies (diluted in 5% normal donkey serum and 0.1% Triton X100) at room temperature in the dark for 2 h. After additional PBS rinses, DAPI (Sigma-Aldrich) counterstain was added. Images were obtained using a motorized, upright fluorescence microscope (Zeiss Axio Imager M2, Carl Zeiss, Oberkochen, Germany). Images were captured using an Imager M2 camera. The mean corrected total cell fluorescence (CTCF) was calculated using ImageJ.

### 2.13. Quantification and Statistical Analysis

For the Western blot analyses, densitometric intensity values were compared using a paired *t*-test for pairwise comparisons. Mammary ductal tree branch lengths were determined utilizing an ImageJ analysis of the captured pictures. Pixel values were transformed into metric values using the provided scale information. Terminal end buds were quantified by manual counting, and the statistical significance was determined using paired *t*-tests for pairwise comparisons between two genotypes. The percent branched organoids were determined by inspecting 50 randomly selected in-Matrigel organoids for each mouse and averaged between the total *n* of each condition. Significance was determined by paired *t*-tests. Hoechst-labeled MEC colonies were counted using captured photographs of the Matrigel pellet. A Muse^TM^ cell analyzer (MilliporeSigma, Burlington, MA, USA) was used to obtain quantitative cell counts. Prior to application of the statistical tests, data distributions were evaluated for normality, and the variance between groups was assessed using Graphpad software. The analysis of the graphs were conducted using Graphpad Prism 5 software. FlowJo (v 8.8.6) was used for all FACS analyses and generation of plots. The specific number of biological replicates are indicated in the relevant figure legends.

## 3. Results

### 3.1. A Role for Rasgrp1 in Mammary Epithelial Cells

**Figure 1:**
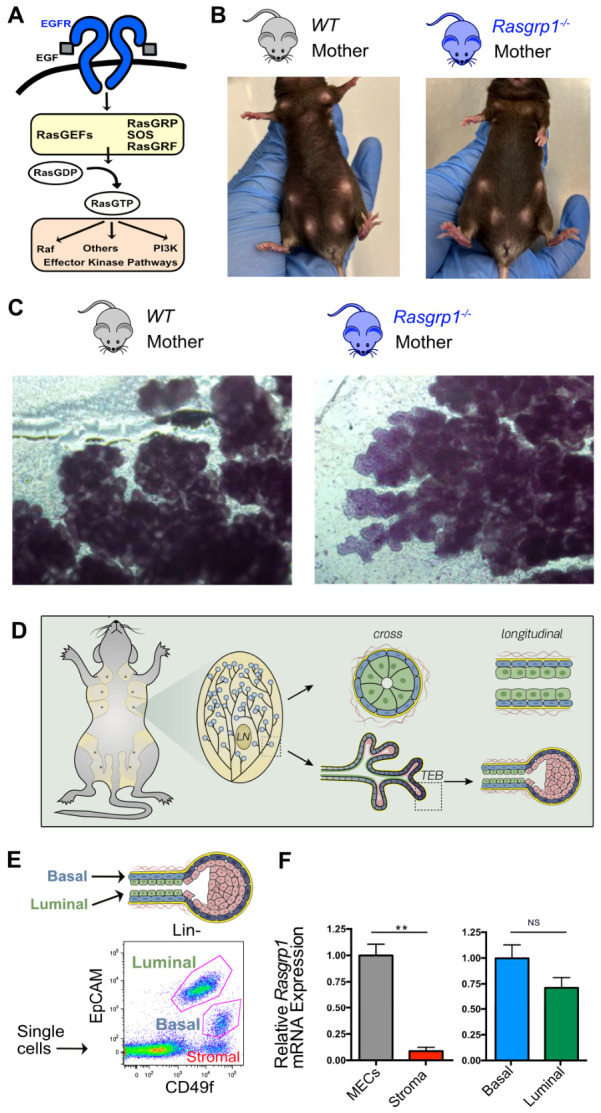
**Rasgrp1 is expressed in the mammary epithelium:** (**A**) schematic of EGFR–RasGEF–Ras-kinase pathways; (**B**) representative images of nursing wild-type (WT) and *Rasgrp1*-deficient mothers; (**C**) Carmine Red stainings of the pectoral mammary gland tissue from nursing wild-type (WT) and *Rasgrp1*-deficient mothers; (**D**) illustration depicting the architecture of the mammary ductal network, duct, and TEB (terminal end bud) make-up; (**E**) sorting on EpCAM and CD49f allows for isolation of basal and luminal MECs; (**F**) Rasgrp1 mRNA expression in MECs. RNA was isolated from total MECs, sorted basal and luminal cells, and the surrounding stromal cells. Taqman RT-PCR was performed for Rasgrp1 transcripts. Expression normalized to β-actin RNA levels. 2^−ddct^ was calculated, with total MECs set to 1. *t*-Tests were performed to determine significance in pairwise comparisons. *n* = 3 for MECs and stroma, *n* = 2 for basal and luminal. (** *p* < 0.005, NS = not significant).

SOS1 and RasGRP1 both activate Ras but display different molecular regulation [16,17,18]. Initially, Rasgrp1 was thought to be a T-cell-specific Ras activator [31]. However, since the report of a T-cell developmental block in *Rasgrp1*-deficient mice, many more expression sites for Rasgrp1 have been reported (reviewed in [24]). In colorectal cancer, Rasgrp1 dampens EGFR–SOS1 signals and downstream RasGTP–ERK signals (Figure 1A) [28], and deletion of *Rasgrp1* leads to accelerated tumor growth in mouse models of colorectal cancer [28,35].

We noted in our mouse colony that pups from *Rasgrp1*-deficient moms remained relatively small until they switched to eating solid food. We crossed WT (wild-type) *C57BL/6* males with WT or *Rasgrp1*-deficient *C57BL/6* females, and the pectoral group of nipples from *Rasgrp1*-deficient moms appeared smaller (Figure 1B), and Carmine Red stainings revealed a reduced density of the mammary gland tissue (Figure 1C). The mammary epithelium consists of basal and luminal layers of epithelial cells, and we sorted mammary epithelial cell (MEC) subpopulations by flow cytometry to perform Taqman analysis for *Rasgrp1* mRNA expression (Figure 1C–E). Rasgrp1 was expressed in MECs but displayed negligible expression in the stroma (Figure 1F). CD31-/CD45-/Ter119- “lineage-negative” cells (excluding endothelial cells, lymphocytes, and erythrocytes) can be divided into basal and luminal cells via analysis of EpCAM and CD49f expression [36,37]. Rasgrp1 expression was detected in both basal and luminal cells (Figure 1F).

### 3.2. Rasgrp1 Dampens EGFR–PI3K Signals in MECs as Well as Single-Cell MEC Colony Growth

**Figure 2:**
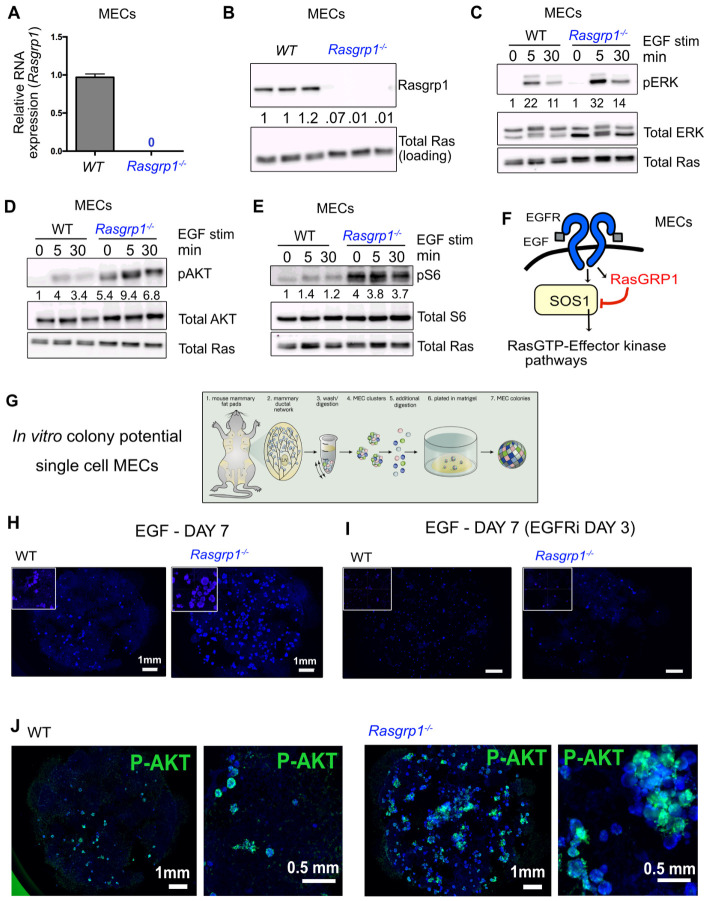
**Rasgrp1 dampens proliferative EGFR–AKT signals in mammary epithelial cells.** (**A**) Rasgrp1 mRNA expression in MECs. RNA from isolated MECs was subjected to Taqman RT-PCR for Rasgrp1 transcripts. Expression was normalized to β-actin RNA levels. *n* = 4 per genotype. (**B**) Rasgrp1 protein expression in wild-type (WT) and Rasgrp1-deficient MECs. Total Ras was used as the loading control. (**C**–**E**) EGF-induced ERK, AKT, and S6 kinase signals in wild-type and Rasgrp1^−/−^ MECs. Western blot detection of phosphorylated ERK-, AKT-, and S6-proteins after 0, 5, and 30 min of EGF stimulation. Total ERK, AKT, and S6 were consistent among the samples. Total Ras served as an additional loading control. Ratiometric densitometry quantification of P-ERK/ERK, P-AKT/AKT, and P-S6/S6 is indicated below the blots with WT 0 stimulation arbitrarily set at 1.0 for all analyses. The blots in (**B**–**E**) are representative examples of the three independent experiments. (**F**) Schematic of Rasgrp1 as a brake on the EGFR–SOS1–Ras pathway in MECs. (**G**) Illustration of the mammary epithelial cell colony-forming assay. Single cells were placed into a Matrigel pellet and monitored for 7 days to assay the colony number and size. (**H**,**I**) *Rasgrp1^−/−^* single MECs formed more numerous and larger clusters in response to EGF compared with WT. In each Matrigel pellet, 2500 cells were loaded. Hoechst 33342 (blue) was used as a nuclear counterstain. Scale bar: 1 mm. Zoomed in images are in the top left. Colony formation potential relies on continued EGFR signaling input. 10 µM Erlotinib (EGFRi = EGFR inhibitor) was added at Day 3. (**J**) AKT phosphorylation of the colony-formation assay on the indicated plated single MEC populations. Scale bar: 1 mm. Images on the right are higher in magnification; scale bar: 0.5 mm.

Having established Rasgrp1 expression in epithelial cells of the mammary gland, we next isolated total MECs from wild-type (WT) or *Rasgrp1*-deficient mice and ran Taqman analyses for *Rasgrp1* mRNA levels (Figure 2A), as well as Western blot analysis for Rasgrp1 protein expression (Figure 2B). As expected, *Rasgrp1*-deficient MECs do not express Rasgrp1 mRNA or protein.

We used these WT and *Rasgrp1*-deficient MECs to test the dynamics of EGF-induced Ras-kinase signals. EGF stimulation of *Rasgrp1^−/−^* MECs resulted in modestly enhanced ERK phosphorylation (P-ERK), a downstream effector of the Ras–RAF–MEK kinase pathway, compared to the quantitated signal in EGF-stimulated wild-type MECs (Figure 2C). As a downstream effector of PI3K signaling, AKT promotes protein synthesis, cell proliferation, cell metabolism, and cell survival [38]. We observed substantially elevated baseline AKT phosphorylation (P-AKT) in *Rasgrp1^−/−^* MECs, which was further induced by EGF stimulation (Figure 2D). ERK and AKT can both connect to the ribosomal S6 signaling pathway, which plays roles in translation and metabolism. *Rasgrp1^−/−^* MECs also displayed robustly increased baseline levels of pS6 compared to the wild type (Figure 2E). To assess the cell biological effects of *Rasgrp1* perturbation in mammary epithelium, we digested the mammary gland with enzymes into single cells and subsequently plated these in the presence of EGF to probe MEC proliferative capacity in sphere-forming assays (Figure 2G). Using this assay, we observed increased colony formation for *Rasgrp1^−/−^* compared to WT MECs (Figure 2H). Both the increased colony formation of *Rasgrp1^−/−^* MECs and the normal potential of WT MECs were dependent on active EGFR signaling (Figure 2I). Immunofluorescence (IF) on the spheres revealed that the larger and more numerous *Rasgrp1^−/−^* colonies displayed high levels of P-AKT (Figure 2J).

In sum, a deficiency in *Rasgrp1* results in increased signals through ERK-, AKT-, and S6-effector kinase pathways in total MECs, and Rasgrp1 functions as a dampener of EGFR-kinase signals in mammary epithelial cells, analogous to its inhibitory role in colorectal carcinoma cells [28,35]. Correspondingly, *Rasgrp1*-deficient MECs display enhanced proliferative capacity in sphere-forming assays.

### 3.3. EGF Potently Impacts Wnt- and R-Spondin-Driven, Stem-like Molecular Programs in Mammary Gland Organoids

**Figure 3:**
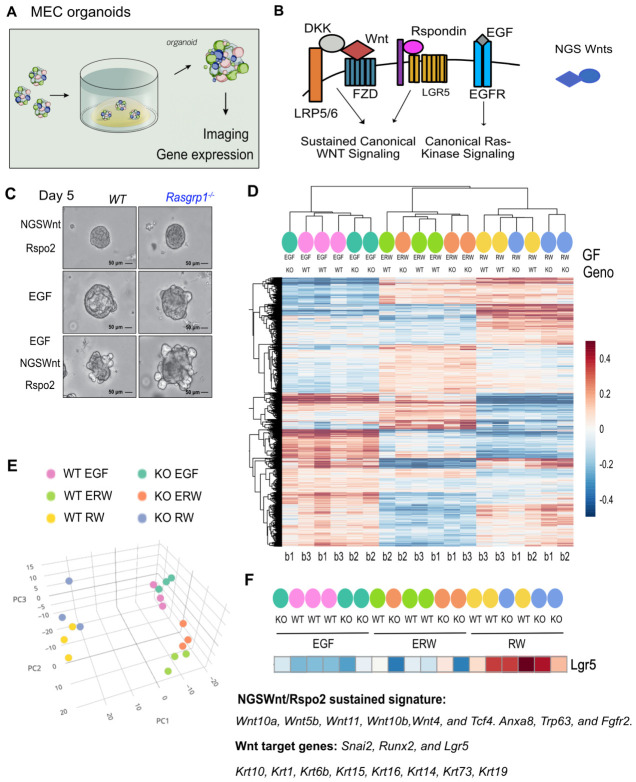
**Rasgrp1 dampens proliferative EGFR–AKT signals in mammary epithelial cells.** (**A**) Illustration of the 3D MEC organoid Matrigel assays. Isolated mammary fat pads are enzymatically digested. Differential centrifugation separates mammary epithelial cell clusters, which grow into 3D structures in Matrigel. (**B**) Illustration of surrogate Wnt ligands (NGS Wnt), R-Spondin (Rspo), and EGF triggering canonical Wnt and Ras–Kinase pathways. Organoids are set up in fully defined medium with precise growth factor concentrations and no conditioned media. (**C**) Morphology of wild-type and *Rasgrp1^−/−^* mammary epithelial organoid assays in response to exposure to the indicated growth factors. Images are captured on day 5. (**D**) Unsupervised clustering of total RNAseq data from wild-type (WT) and *Rasgrp1^−/−^* (KO) mammary epithelial organoid assays at day 5. Each RNA sample is a pool of two age-matched littermates. The experiment was performed in three batches (b1–b3). The significance cut-off for the false discovery rate < 0.05. No fold-change thresholding was used to restrict genes in 3D. Genotype and RW, ERW, and EGF growth factors are indicated. Raw data were deposited at GEO NCBI. (**E**) Principal component analysis was conducted on the eighteen generated gene signatures. Color-coding is the same as in (**D**). (**F**) Summary of the gene expression maintained by RW but lost by the addition of EGF. Examples include several Wnt pathway components, Wnt signal modifiers, and Wnt target genes such as *Lgr5*. For a complete list see Supplementary Figure S1.

Three-dimensional (3D) cultures of mammary epithelium have provided important insights in the complex biology of this organ [39] (Figure 3A). Inclusion of Wnt3a and Rspo2 (R-spondin 2) in growth media facilitates organoid formation and budding that grow out to structures with internal, polarized luminal cells and an outer network of elongated myoepithelial cells [40]. Wnt signals support stem cell function in many organs and enable efficient generation of organoids in Matrigel in vitro [41,42]. Binding of the ligand R-spondin to the receptor Lgr5 (Leu-rich repeat-containing receptor 5) sustains Wnt signals [43]. Design and generation of surrogate Wnt ligands (DKK Dickkopf, combined with Wnt, Figure 3B) used in tandem with R-spondin triggers Lgr5 and LRP/Frizzled receptors to mimic sustained, canonical Wnt signaling and enables effective growth of organoids [44]. “Next-generation surrogate” (NGS) Wnts (Figure 3B) are the next version and have proven highly effective to initiate and expand organoids from multiple different types of tissues, including kidney, colon, pancreas, hepatocyte, ovarian, and breast [45]. Using colon organoids, we performed careful titering of the Wnt signals, and as little as 0.1 nM of NGS Wnt sustained colon organoids [45].

To understand the impact of defined growth factor environments on WT and *Rasgrp1^−/−^* mammary epithelial cells, we plated MECs in well-defined, simple DMEM/F12 medium with insulin–transferrin–selenium and systematically assessed the impact of added NGS Wnt/Rspo2 (RSpondin2), EGF, or the combination of Wnt/Rspo2/EGF on generated organoids. Titrating NGS Wnt and Rspo2 revealed that 0.1 nM and 8.3 nM, respectively, sustained healthy growth while allowing for additional cell biological effects induced by 2.5 nM EGF. We purposely used the most basic media here in order to focus on the LRP5/6/Frizzled, Lgr5, and EGFR signals, without adding components such as FGF7, FGF10 and Noggin, which are described for breast cancer organoids [46], nor any conditioned media components. Morphologically, both WT and *Rasgrp1^−/−^* MEC organoids demonstrated robust budding when exposed to the combination of EGF-, Wnt-, and R-spondin signals (“ERW”) (Figure 3C).

We next defined transcriptional programs driven by EGF, Wnt, and R-spondin, alone or in combination, by performing bulk RNA-seq on day 5 organoids. Unsupervised clustering showed that growth factor conditions dominated gene expression patterns, outweighing genotype (WT vs. *Rasgrp1^−^/^−^*) (Figure 3D and Supplemental Figure S1). RW and EGF conditions were most distinct, with ERW intermediate but not simply additive. Principal component analysis confirmed this: PC1 (44% variance) separated RW from EGF/ERW, with PC2–3 adding resolution (Figure 3E). We hypothesized that RW conditions maintain stem-like programs, while EGF shifts cells away from these states.

Consistent with this model, a gene cluster enriched in RW and reduced in ERW/EGF (Figure 3F) showed elevated expression of Wnt target genes, such as *Snai2* and *Runx2*, and *Lgr5*, the receptor for R-spondin [47,48,49,50,51], supporting active Wnt signaling under RW conditions. The RW organoid-specific gene cluster also contained multiple *Wnt* ligands, indicating active autocrine Wnt signaling, and expressed the transcription factor *Tcf4*, which is known to associate with beta-catenin to turn on target genes in response to Wnt signals [52]. Third, this cluster included Wnt modulators (*Anxa8*, *Trp63*, and *Fgfr2*), all linked to β-catenin signaling or epithelial progenitor function [53].

Lastly, whereas specific growth factor combinations clearly dominate gene expression profiles, we also explored the unique differences between organoids from WT and *Rasgrp1^−/−^* MECs for completeness. These analyses resulted in a limited list of genes expressed at lower levels or higher levels in *Rasgrp1^−/−^* MECs (Supplemental Figure S2).

### 3.4. Rasgrp1 Deficiency Impacts Coordinated Mammary Ductal Tree Formation

During puberty, the mature mammary ductal tree is formed; bulbous TEBs form at the tips of ducts and coordinated cell proliferation, and migration drives the invasion of TEBs into the fat pad [1,2,3,4]. Branching is critical here, either through TEB bifurcation or via secondary side-branch sprouting.

**Figure 4:**
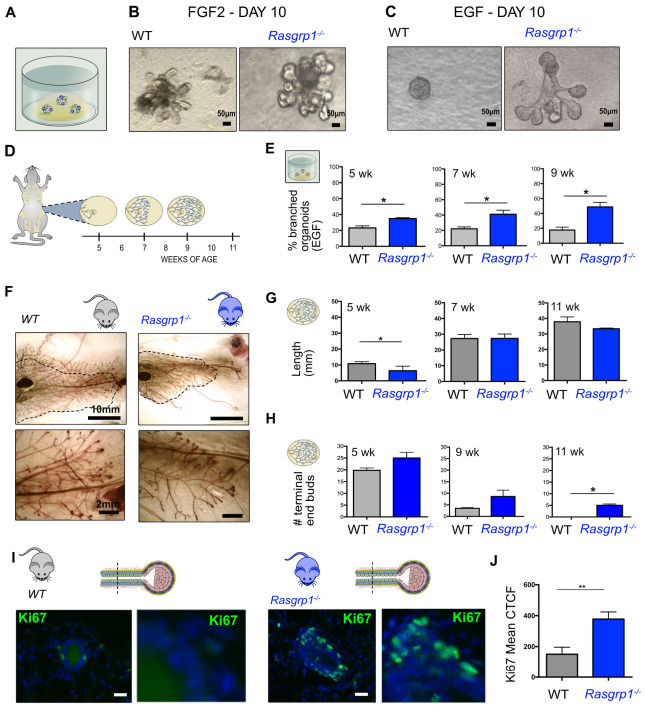
**Cell biological features of Rasgrp1 deficiency in MEC organoids and the mammary gland.** (**A**) Organoid approaches. (**B**) Three-dimensional cultures derived from WT or *Rasgrp1^−/−^* mice all respond robustly to the branching agonist FGF2. Representative images are shown for FGF2-stimulated branched 3D cultures derived from 7-week-old mice, imaged at day 10. Scale bar: 50 μm. (**C**) *Rasgrp1^−/−^*-derived 3D cultures had augmented branching in response to EGF stimulation. Representative 3D culture images are shown for each group, derived from 7-week-old mice, imaged at day 10. Scale bar: 50 μm. (**D**) Illustration depicting pubertal mammary gland development in C57BL/6 mice. Shown is the #4 inguinal fat pad. Onset of pubertal development from the primitive duct occurs at 5 weeks, reaches an approximate midpoint at 7 weeks, and concludes at 9 weeks of age. (**E**) *Rasgrp1^−/−^*-derived 3D cultures of 5-, 7-, and 9-week-old mice all have augmented branching in response to EGF stimulation (*t*-test * *p* < 0.05, *n* = 4 for each genotype). (**F**) Seven-week-old mammary whole mounts from WT and *Rasgrp1^−/−^* mice were carmine stained and imaged on a stereo dissection scope. Scale bar: 10 μm. (**G**) Quantification of ductal elongation. Length was measured from the nipple-proximal end of the lymph node to the furthest epithelial branch. At early pubertal development, *Rasgrp1^−/−^* mice displayed significantly shorter ductal elongation (* *p* < 0.05; *n* = 4). Statistical significance was determined by *t*-test. (**H**) *Rasgrp1^−/−^* mice showed an elevated number of proliferative epithelial structures and terminal end buds (TEBs) at week 11. Statistical significance was determined by *t*-test. (* *p* < 0.05; *n* = 4). (**I**) Ki67 immunofluorescence (green) on cross-sections of terminus proximal mammary ducts from 9-week-old WT or *Rasgrp1^−/−^* mice. DAPI (blue) counterstaining marks the cell nuclei. Representative examples are shown. Scale bar: 10 μm. Bottom images are a higher magnification. (**J**) Quantification of the mean corrected total cell fluorescence (CTFC) for Ki67 IF. *n* = 4 for both groups. Significance was determined by *t*-test. (** *p* < 0.005).

FGF2 is known to potently promote branching of MECs in mammary organoids (Figure 4A) over the course of 10 days [39,54]. As expected, FGF2 stimulated robust branching in 60 to 80% of our 3D Matrigel cultures from both genotypes (Figure 4B). Normally, EGF only induces spherical growth without substantial branching [55], but *Rasgrp1^−/−^* MEC organoids demonstrated substantial branching when stimulated solely with EGF by 10 days of culture (Figure 4C), indicative of a perturbed cell biological program. To assess that the aberrant branching was not a reflection of an altered developmental stage, we generated MEC organoids from 5-, 7-, and 9-week-old C57BL/6 mice (Figure 4D); all *Rasgrp1^−/−^* organoids displayed altered branching patterns in the presence of EGF (Figure 4E).

Lastly, to assess the impact of *Rasgrp1* deficiency on the ductal phase during puberty in vivo, we analyzed the morphology of the ductal tree from 5-, 7-, 9-, and 11-week-old C57BL/6 mice to capture pubertal development stages. Rasgrp1^−/−^ females revealed disrupted ductal tree morphology with a runted appearance (Figure 4F), decreased length at week 5 (Figure 4G), and increased TEB numbers at 11 weeks (Figure 4H). The presence of TEBs in 11-week-old *Rasgrp1^−/−^* females is remarkable, as these structures have normally vanished by this age. At 9 weeks, sectioned ducts from *Rasgrp1^−/−^* females display continued proliferation at duct locations that are supposed to contain fully differentiated cells, as evidenced by the high number of Ki67-positive, proliferative cells. By contrast, proliferation had decreased at that age in wild-type females (Figure 4I,J).

## 4. Discussion

Our study here revealed an unanticipated role for Rasgrp1 in supporting coordinated mammary gland development.

*Rasgrp1* perturbation leads to heightened EGFR–Ras–PI3K–AKT and mTORC1-S6 kinase signaling, accompanied by increased proliferative and branching capacity in response to EGF. These findings underscore the necessity for tight quantitative control of EGFR signaling during mammary morphogenesis, consistent with prior studies demonstrating that both insufficient and excessive EGFR activity disrupt development. For example, expression of a dominant-negative EGFR under the MMTV promoter impairs pubertal ductal elongation and reduces epithelial proliferation [56], whereas *Waved-2* mice harboring a hypomorphic EGFR kinase mutation [57] exhibit diminished branching and defective ductal invasion into the fat pad during puberty [8,58].

In contrast, *Rasgrp1* deficiency does not simply enhance overall growth. Instead, it produces a qualitatively distinct phenotype characterized by aberrant epithelial proliferation within ducts and persistence of terminal end buds (TEBs), without a corresponding increase in total mammary tree length or overall cellularity. This dissociation between local proliferative activity and global morphogenetic output suggests a breakdown in the spatial and temporal coordination of mammary gland development. Rasgrp1, therefore, appears to be essential not for driving growth per se but for ensuring that proliferative and morphogenetic programs are properly aligned during pubertal expansion.

Several non-mutually exclusive mechanisms may underlie this loss of coordination in *Rasgrp1*-deficient tissue. First, our organoid studies demonstrate that EGF signaling can strongly suppress NGS Wnt/R-spondin2-driven stem-cell gene programs. Elevated EGFR signaling in *Rasgrp1*-deficient mammary stem or progenitor cells may, therefore, prematurely extinguish Wnt-dependent stem cell states, forcing cells out of a self-renewing program before appropriate morphogenetic cues are completed. This would be consistent with the observed imbalance between proliferation and organized ductal extension.

Second, the marked increase in PI3K–AKT signaling may perturb lineage specification. PI3K pathway activity has been implicated in regulating basal versus luminal cell fate decisions, and inducible expression of the oncogenic *PI3K^H1047R^* allele can drive lineage plasticity and switch the cell fate from luminal to basal and vice versa. In this context, elevated PI3K signaling in *Rasgrp1*-deficient cells may disrupt the fidelity and timing of lineage commitment, leading to inappropriate or asynchronous epithelial tree development.

Together, these findings support a model in which Rasgrp1 functions as a signaling rheostat that integrates EGFR, Wnt, and PI3K pathway activity to coordinate stem cell maintenance, lineage progression, and morphogenesis. Future studies will be required to define the precise molecular mechanisms by which Rasgrp1 modulates pathway crosstalk and to determine whether similar regulatory principles operate in other epithelial tissues or in oncogenic contexts.

## Data Availability

Raw data are deposited at GEO NCBI: GSE 149275 (https://www.ncbi.nlm.nih.gov/geo/query/acc.cgi?acc=GSE149275 (accessed on 14 July 2026)).

## Supplementary Materials

The following supporting information can be downloaded at: https://www.mdpi.com/article/10.3390/cells15141288/s1, Figure S1: Organoid Gene expression data—Wnt and Rsponin2-driven stem cell signatures. Overview of gene expression assessed through RNAseq of organoids. Highlighted in S1 are genes that are expressed at high levels in RW medium but are expressed at lower levels in ERW or EGF; Figure S2: Organoid Gene expression data—Differences WT and Rasgrp1^−/−^ organoids. Overview of gene expression differences by genotype, assessed through RNAseq of organoids.

## Abbreviations

The following abbreviations are used in this manuscript:

EGFR	Epidermal growth factor receptor
EGF	Epidermal growth factor
TGFα	Transforming growth factor-α
AREG	Amphiregulin
MECs	Mammary epithelial cells
TEBs	Terminal end buds
RasGEFs	Ras guanine nucleotide exchange factors
SOS1	Son of Sevenless 1
RasGRP1	Ras-guaninyl-releasing protein 1
PI3K	Phosphatidylinositol-3 kinase
P-ERK	ERK phosphorylation
P-AKT	AKT phosphorylation
UCSF	University of California, San Francisco
WT	Wild type
3D	Three-dimensional
NGS	Next-generation surrogate
