# Supplementary material for: Rasgrp1-Mediated Dampening of EGFR Signals Supports Coordinated Mammary Gland Development
Source: Cells. 2026 Jul 18;15(14):1288. doi: 10.3390/cells15141288 (PMC13406350; doi:10.3390/cells15141288)
Supplement: Supplementary file 1 [file cells-15-01288-s001.zip › cells-4328561-supplementary.pdf]

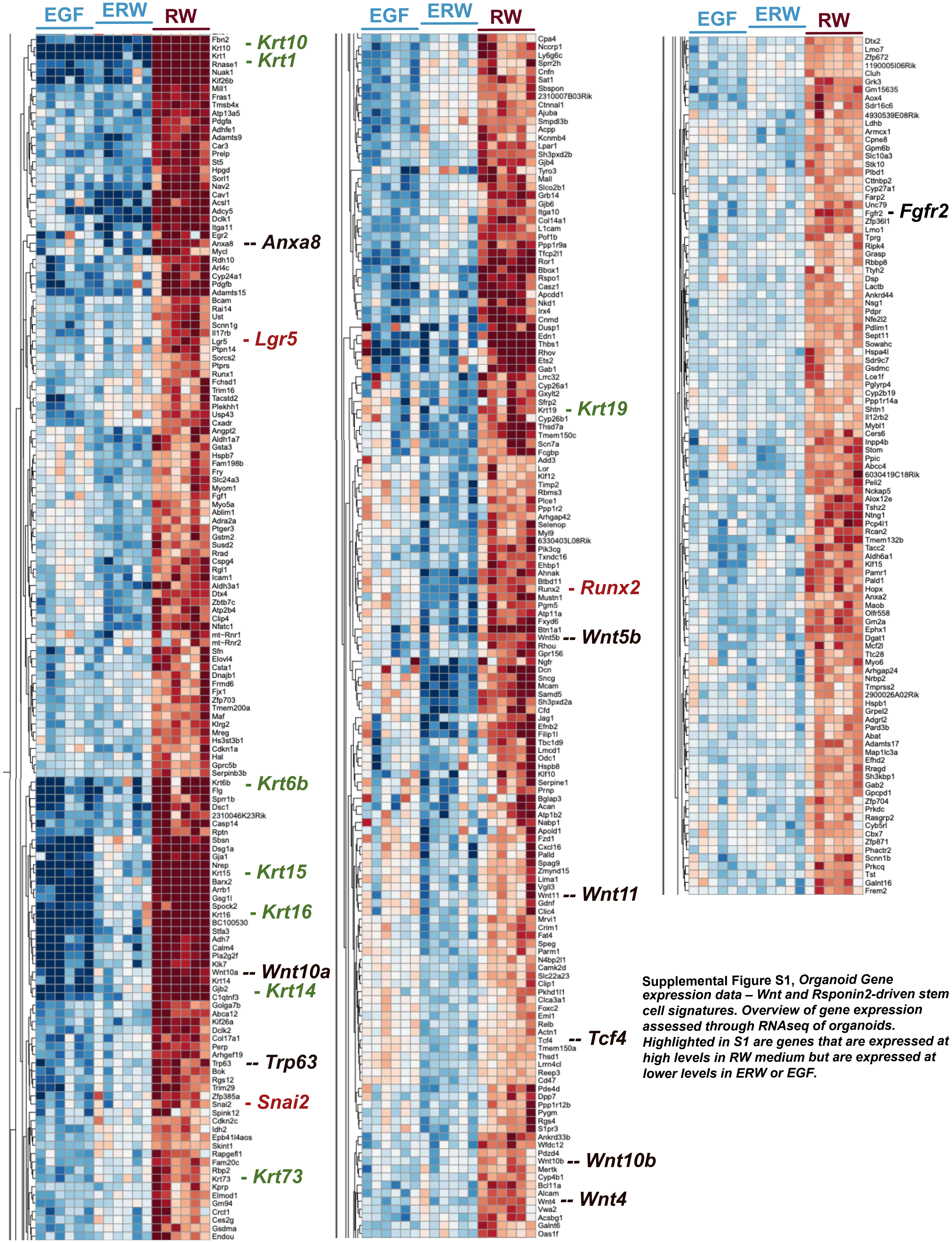

Supplemental Figure S1, *Organoid Gene expression data – Wnt and Rsponin2-driven stem cell signatures. Overview of gene expression assessed through RNAseq of organoids. Highlighted in S1 are genes that are expressed at high levels in RW medium but are expressed at lower levels in ERW or EGF.*

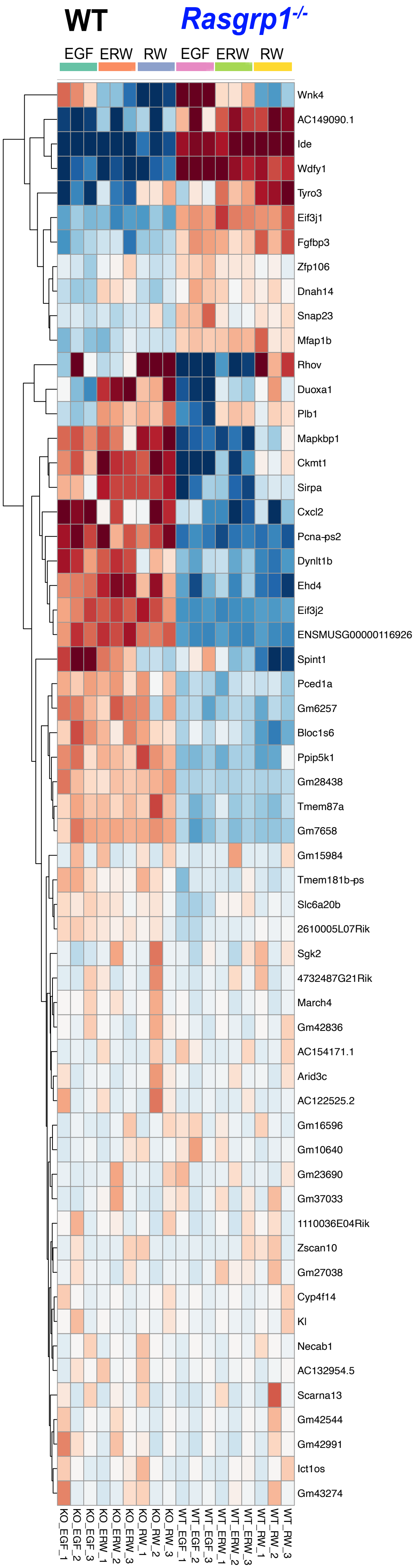

Supplemental Figure S2, ***Organoid Gene expression data – Differences WT and Rasgrp1-/- organoids. Overview of gene expression differences by genotype, assessed through RNAseq of organoids***
